# Activation of Peracetic Acid with CuFe_2_O_4_ for Rhodamine B Degradation: Activation by Cu and the Contribution of Acetylperoxyl Radicals

**DOI:** 10.3390/molecules27196385

**Published:** 2022-09-27

**Authors:** Chengzhi Yu, Libin Zheng, Yongyuan Hong, Jiabin Chen, Feng Gao, Yalei Zhang, Xuefei Zhou, Libin Yang

**Affiliations:** 1 State Key Laboratory of Pollution Control and Resource Reuse, College of Environmental Science and Engineering, Tongji University, Shanghai 200092, China; 2 Shanghai Institute of Pollution Control and Ecological Security, Tongji University, Shanghai 200092, China

**Keywords:** peracetic acid (PAA), rhodamine B, copper ferrite (CuFe_2_O_4_), organic radicals (R-O˙)

## Abstract

Advanced oxidation processes (AOPs) demonstrate great micropollutant degradation efficiency. In this study, CuFe_2_O_4_ was successfully used to activate peracetic acid (PAA) to remove Rhodamine B. Acetyl(per)oxyl radicals were the dominant species in this novel system. The addition of 2,4-hexadiene (2,4-HD) and Methanol (MeOH) significantly inhibited the degradation efficiency of Rhodamine B. The ≡Cu^2+^/≡Cu^+^ redox cycle dominated PAA activation, thereby producing organic radicals (R-O˙) including CH_3_C(O)O˙ and CH_3_C(O)OO˙, which accounted for the degradation of Rhodamine B. Increasing either the concentration of CuFe_2_O_4_ (0–100 mg/L) or PAA (10–100 mg/L) promoted the removal efficiency of this potent system. In addition, weakly acid to weakly alkali pH conditions (6–8) were suitable for pollutant removal. The addition of Humid acid (HA), HCO_3_^−^, and a small amount of Cl^−^ (10–100 mmol·L^−1^) slightly inhibited the degradation of Rhodamine B. However, degradation was accelerated by the inclusion of high concentrations (200 mmol·L^−1^) of Cl^−^. After four iterations of catalyst recycling, the degradation efficiency remained stable and no additional functional group characteristic peaks were observed. Taking into consideration the reaction conditions, interfering substances, system stability, and pollutant-removal efficiency, the CuFe_2_O_4_/PAA system demonstrated great potential for the degradation of Rhodamine B.

## 1. Introduction

With the increasing maturity of activated sludge processing technologies and physicochemical treatment methodologies, common pollutants present in sewage represented by parameters such as the chemical oxygen demand (COD), nitrogen content, and phosphorus content have been effectively treated. However, there are still problems associated with the removal of micropollutants with complex structures, which has spurred great research interest in recent years. Due to the appreciable removal performance of persistent organic pollutants in sewage effluent and industrial wastewater, advanced oxidation processes (AOPs) have attracted more and more attention and have been considered one of the most promising technical treatment options [1,2,3]. AOPs consist of various technologies, such as ozonation, photocatalytic oxidation, Fenton-like oxidation, electrochemical oxidation, etc. The basis for these treatment methodologies relies on reactive oxygen species (ROS) such as hydroxyl radicals, sulfate radicals, and organic radicals [4,5,6].

Several oxidants such as hydrogen peroxide (H_2_O_2_), ozone (O_3_), persulfate (PDS), and peroxymonosulfate (PMS) are used in AOPs to generate free radicals [7,8,9]. Hydrogen peroxide is the most-used oxidant because of the high activity of its oxidizing species (e.g., HO˙). Moreover, these oxidizing species are capable of completely and non-selectively destroying recalcitrant organic contaminants [10]. Recently, the application of peracetic acid (PAA) in AOPs has been studied. PAA, a peroxy acid oxidant, has been widely used for disinfection and sterilization in textile industries, aquaculture, medical applications, and food processing [11,12]. Owing to its low toxicity, less harmful byproducts, and high disinfection efficiency, the application of PAA has increased continuously as an alternative disinfectant in wastewater treatment systems in Europe and North America. The standard reduction potential of PAA (relative to the standard hydrogen electrode) is 1.06–1.96 V, which is close to that of H_2_O_2_ (E_0_ = 1.8 V) [11]. However, the peroxide bond energy of PAA is 159 kJ/mol, which is lower than that of hydrogen peroxide (HP, 213 kJ/mol) and PMS (317 kJ/mol) [13,14], indicating that PAA can be activated more easily to produce oxidative radicals. Moreover, PAA has less dependence on pH, and its decomposition products are non-toxic, safe, and biodegradable [15,16,17,18,19]. When integrated with biotreatment, these decomposition products can supply additional carbon sources [20]. In summary, PAA is an ideal novel oxidant; however, developing an efficient and pollution-free activation method of PAA is of great significance.

Many researchers have devoted significant time to exploring efficient catalysts for PAA activation, and considerable progress has been made on this front. In AOPs with PAA as the oxidant, degradation of persistent organic pollutants is driven by various radicals, including OH˙ and R-C˙ (e.g., CH_3_˙, CH_3_O_2_˙, CH_3_CO˙, CH_3_CO_2_˙, and CH_3_CO_3_˙) [21,22]. Thus, the catalytic decomposition of PAA is more complicated compared with the activation of inorganic peroxides. Transition metals, ultraviolet/solar light irradiation, ultrasound, heat, electrochemical processes, and carbon catalysts are all techniques used in AOPs [22,23,24]. The techniques that have been most applied for PAA catalysis are UV radiation and the inclusion of transition metals. The dominant radicals may be different when different activation systems are applied. In the UV/PAA system, HO˙ and CH_3_CO_2_˙ are the initial products (Equation (1)). Subsequently, CH_3_CO_3_˙ is generated (Equation (2)) [25].

CH_3_CO_3_H + UV ⇌ HO˙ + CH_3_CO_2_˙ (1)

CH_3_CO_3_H + HO˙ → CH_3_CO_3_˙ + H_2_O (2)

As for the transition metal/PAA system, the products are much more complex (Equations (3)–(8)), among which CH_3_CO_2_˙ and CH_3_CO_3_˙ are regarded as the dominant radicals, while little to no HO˙ is generated [26,27].

CH_3_C(O)OOH + M^n+^ → M^(n+1)+^ + HO^−^+ CH_3_COO˙ (3)

CH_3_C(O)OOH + M^(n+1)+^ → M^n+^ + H^+^ + CH_3_COOO˙ (4)

(5) $${\mathrm{CH}}_{3}C\left(O\right)\mathrm{OOH}\;\overset{M^{n+}}{\to }{\;\mathrm{CH}}_{3}C\left(O\right)O˙+\;\mathrm{HO}˙$$

CH_3_C(O)OOH + HO˙ → CH_3_C(O)OO˙ + H_2_O (6)

CH_3_C(O)O˙ → CH_3_˙ + CO_2_ (7)

CH_3_˙ + O_2_ →CH_3_O_2_˙ (8)

The different relative species that contributed to contaminant degradation were further probed in some studies. For example, it has been reported that CH_3_CO_2_˙ and CH_3_CO_3_˙ demonstrated a good organic compound removal efficiency for compounds such as naproxen, naphthyl compounds, phenol, 2-naphthoxyacetic acid, aniline, bezafibrate, acid orange 7, bisphenol-A, carbamazepine, diclofenac, ibuprofen, clofibric acid, methylene blue, sulfa drugs, and sulfamethoxazole. Furthermore, these radicals had a longer half-life than HO˙ [28,29]. Zhou et al. [16] reported that in the PAA/activated carbon fiber system for the degradation of red X-3B, HO˙ was not important compared to acetyl(per)oxyl radicals. Kim et al. [17] delineated that in the biphasic kinetics of Fe(II)/PAA contaminant degradation, acetyl(per)oxyl radicals, HO˙, and Fe(IV) were responsible reactive species for the degradation of contaminants. 

Transition metal/PAA systems may demonstrate greater promise in the future. However, toxicity and poor reusability bring hindrances to the application of transition metals in homogeneous processes [30]. To overcome these shortcomings, heterogeneous catalysis could be a good alternative. Yu et al. [31] successfully applied fabricated magnetically separable TiO_2_-FeO_x_-polyoxotungstate (POM) to 2,4-DCP degradation, which exhibited excellent performance. Karbasi et al. [32] studied the use of magnetized photocatalysts (Bi_2_WO_6_-FeO_x_) to avoid the high separation cost of catalyst recycling. An environmentally benign and non-toxic catalyst for PAA activation is desirable based on sustainable and green development. 

Recently, spinel-type particles with the general formula MFe_2_O_4_ (M = Cu, Zn, Co, Mn, etc.) have attracted great interest [33]. Spinel ferrite, a typical spinel ferrite, has a relatively stable structure, which reduces the leaching of heavy metals [34]. Another advantage of spinel ferrite is its magnetic property; thus, it can be easily separated from water for recovery [35]. Moreover, the spinel oxide powders could provide a larger surface area to assist the diffusion of reactants onto the active sites. Although CoFe_2_O_4_ has a better catalytic ability than CuFe_2_O_4_, the release of Co during the reaction may bring more risk of toxicity and carcinogenicity [36]. In recent studies, the PMS/CuFe_2_O_4_ system has been explored for the degradation of bisphenol-A at neutral pH values [37], and CuFe_2_O_4_ has been used to generate SO4˙ from PMS [38].

As a synthetic xanthene dye, Rhodamine B was often chosen as a target containment to investigate various AOPs systems [39,40,41]. In the present work, the degradation of Rhodamine B by peracetic acid activated with a spinel copper ferrite (CuFe_2_O_4_) was studied. The morphology, elemental composition, total pore volume, and BET surface area of the catalyst were systematically investigated. The influence of different pH conditions, PAA, and catalyst loading on the pollutant removal efficiency was explored. Furthermore, the activation mechanism and reactive species were further proposed. Finally, the effect of water matrices, reusability, and stability of the catalyst was discussed to see if the PAA/CuFe_2_O_4_ demonstrated a practical utilization value.

## 2. Results and Discussion

### 2.1. Characterization of the Catalyst

**Figure 1:**
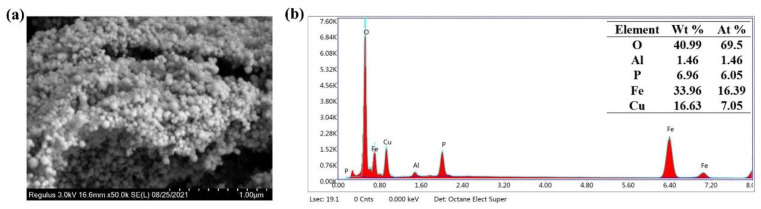
SEM image (**a**) and EDS spectroscopy (**b**) of CuFe_2_O_4_.

To understand the microstructure of the commercial catalyst, the surface morphology of CuFe_2_O_4_ was studied via SEM. As shown in Figure 1a, CuFe_2_O_4_ possessed a relatively regular shaped particle morphology. All catalysts contained small particles with even surfaces. The particles agglomerated together due to the magnetic properties of the material, which was consistent with previous findings. Furthermore, the elemental compositional analyses of the catalysts were conducted by Energy Dispersive X-ray Spectroscopy (EDS). As shown in Figure 1b, five elements were present in the commercial sample: O, Al, P, Fe, and Cu. The atomic contents of these elements were 69.5%, 1.46%, 6.05%, 16.39%, and 7.05%, respectively. The total pore volume of CuFe_2_O_4_ was 0.067 cm^3^/g, and the BET surface area of CuFe_2_O_4_ was 19.4 m^2^/g.

### 2.2. The Effect of Initial pH on Contaminant Degradation

**Figure 2:**
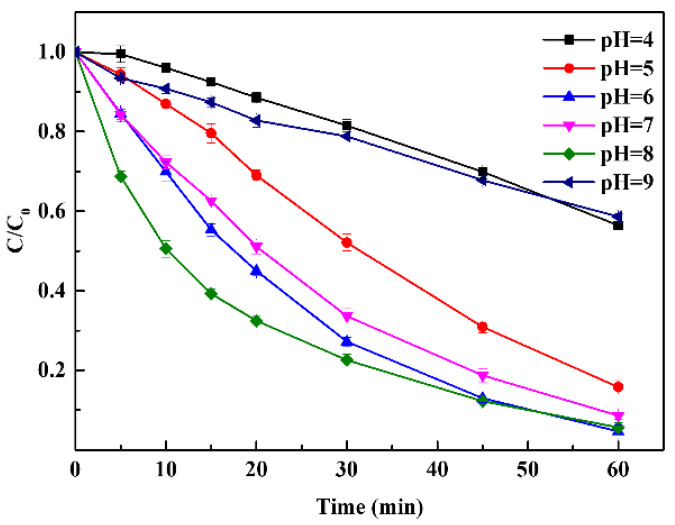
Effect of pH on Rhodamine B degradation (CuFe_2_O_4_ = 100 mg/L, PAA = 40 mg/L, C_0_ = 20 mg/L, room temperature for 1 h).

In metal-based catalysis, the solution pH could affect the oxidation potential of a process, which is considered a crucial parameter for chemical oxidation reactions. The effect of initial pH on the degradation of Rhodamine B in the CuFe_2_O_4_/PAA system was further evaluated, as shown in Figure 2. Different degradation efficiencies could be observed at different pH values. At pH 4 and 9, the removal efficiencies of the system were 18.5% and 21.2% within 30 min, respectively. The sequence of degradation efficiencies under other selected pH values was as follows: pH 5 (47.9%) < pH 7 (66.4%) < pH 6 (72.8%) < pH 8 (77.5%). After 60 min of reaction, the sequence of degradation efficiencies changed to the following: pH 9 (41.4%) < pH 4 (43.6%) < pH 5 (84.3%) < pH 7 (91.4%) < pH 8 (94.4%) < pH 6 (95.4%). It can be inferred that the specific pH range (weakly acid to weakly alkaline conditions, including neutral conditions) was favorable for Rhodamine B decomposition. When the pH condition was higher than eight or lower than six, the removal efficiency decreased sharply. There were two possible causes for this phenomenon: the first was the abnormal decomposition of PAA and the second was the change of the catalyst state. For acidic conditions, it has been reported that the structure of PAA could remain stable under pH 3–5 [1]. In addition, PAA activation by Cu^2+^ could be suppressed under acidic conditions in AOP systems because high concentrations of H^+^ could inhibit the generation of CH_3_CO_3_˙ via Equation (9). Therefore, the degradation of Rhodamine B would be suppressed. Conversely, the causes changed for alkaline conditions. It has been reported in previous studies that the pKa of PAA was 8.2 [42], indicating that the self-decomposition of PAA can easily occur to produce CH_3_CO_3_^−^ when the pH was 9 or greater. Moreover, PAA was a precursor for H_2_O_2_ according to Equation (10) [43]. Therefore, the generation of CH_3_CO_3_^−^ and the reaction between H_2_O_2_ and CH_3_CO_3_^−^ via Equation (11) caused the reduction of PAA, which affected the generation of radicals due to the lack of substrate activation. In terms of the catalyst, the high OH^−^ concentration could also suppress the generation of radicals via Equation (12). Moreover, the Cu(OH)_2_ complex formed under alkaline conditions through the reaction between CuFe_2_O_4_ and OH^−^ [1], which had a lower solubility and lower activity than free ions. In heterogeneous systems, these complexes are attached to the catalyst surface easily [44]. Therefore, the PAA activation efficiency was reduced by the reduction of both the catalyst and the contact area. In summary, the results showed that weakly acidic to weakly alkaline pH conditions were all suitable for PAA degradation in the CuFe_2_O_4_/PAA system. For actual sewage treatment (e.g., domestic sewage, livestock and poultry breeding wastewater, and some kinds of industrial wastewater), the CuFe_2_O_4_/PAA system holds practical value, without the need for excess acid and alkaline conditions.

Cu^3+^ + CH_3_C(O)OOH ⇌ Cu^2+^ + CH_3_C(O)OO˙ + H^+^ (9)

CH_3_C(O)OO H + H_2_O ⇌ H_2_O_2_ + CH_3_C(O)OH (10)

CH_3_C(O)OO^−^ + H_2_O_2_ → CH_3_C(O)OO^−^ + H_2_O + O_2_ (11)

CH_3_C(O)OO H + Cu^2+^ → Cu^3+^ + OH^−^ + CH_3_C(O)O˙ (12)

### 2.3. The Effect of PAA and Catalyst Loading on Contaminant Degradation

**Figure 3:**
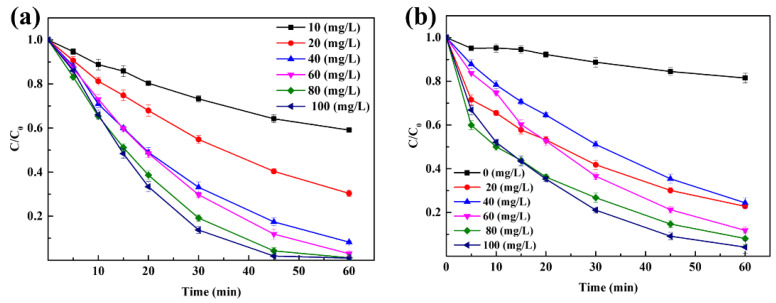
(**a**) Effect of PAA loading on Rhodamine B degradation (CuFe_2_O_4_ = 100 mg/L, pH = 7, C_0_ = 20 mg/L, room temperature for 1 h). (**b**) Effect of catalyst loading on Rhodamine B degradation (PAA = 80 mg/L, pH = 7, C_0_ = 20 mg/L, room temperature for 1 h).

The degradation of Rhodamine B under different PAA concentrations and catalyst loadings in the CuFe_2_O_4_/PAA system at pH 7 was explored as summarized in Figure 3.

Figure 3a shows that the degradation of Rhodamine B was significantly enhanced, as the PAA concentration increased when 100 mg/L CuFe_2_O_4_ was added. When the PAA concentration increased from 10 mg/L to 100 mg/L, the degradation of Rhodamine B in the CuFe_2_O_4_/PAA system after 60 min increased from 40.9% to 99.0%. The results indicated that a higher PAA concentration produced more active radicals at the same time interval, which could accelerate the degradation of Rhodamine B. When the PAA concentration was 60 mg/L, the degradation efficiency of Rhodamine B after 60 min was 97.0%, while a higher degradation efficiency (98.1%) was achieved in a shorter time (45 min) when the PAA concentration was 100 mg/L, which also supported the aforementioned finding. However, the removal efficiency reached 95.8% after 60 min when the PAA concentration was 80 mg/L; thus, further increasing the PAA loading was not conducive to controlling cost. In addition, negligible degradation of Rhodamine B was observed in the absence of PAA, illustrating that the adsorption of Rhodamine B by CuFe_2_O_4_ could be ignored (Figure S1).

Figure 3b demonstrates that the degradation of Rhodamine B was significantly enhanced as the CuFe_2_O_4_ concentration increased when 80 mg/L PAA was added. In the absence of CuFe_2_O_4_, only 18.5% of Rhodamine B was degraded after 60 min, which indicated that the degradation of pollutants mainly depended on the presence of active species, and the oxidative degradation effect by PAA alone was relatively poor. When the CuFe_2_O_4_ concentration increased to 100 mg/L, the degradation of Rhodamine B in the CuFe_2_O_4_/PAA system after 60 min increased to 95.8%. The results showed that an increase in the CuFe_2_O_4_ concentration could provide ample active sites for PAA to generate more free radicals during the same time interval. In addition, as a kind of magnetic material, CuFe_2_O_4_ could be successfully separated by an external magnetic field. Therefore, a higher catalyst loading was acceptable even if cost control was taken into consideration. This also suggested that CuFe_2_O_4_ had broader application prospects than other reported heterogeneous metallic catalysts for the treatment of aquatic micro-organic pollutants.

### 2.4. Identification of Activation Metal

**Figure 4:**
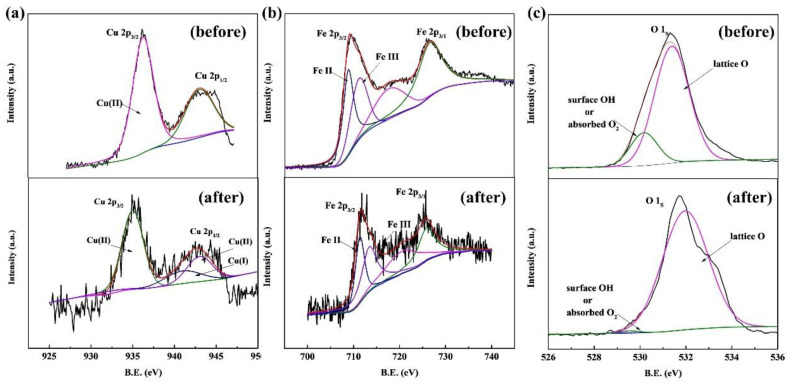
XPS spectra of CuFe_2_O_4_. (**a**) Cu. (**b**) Fe. (**c**) O.

The XPS spectra of Cu, Fe, and O in the CuFe_2_O_4_/PAA system before and after Rhodamine B degradation were obtained to illustrate the catalytic mechanism. The full-scale XPS spectrum of CuFe_2_O_4_ (the binding energies were corrected by the C 1s peak) is shown in Figure S2, which demonstrated the presence of Cu, Fe, and O. Other details are presented in Figure 4.

Figure 4a shows the XPS spectrum of Cu 2p before and after the reaction. The binding energy peaks at 942.70 eV and 935.30 eV could be ascribed to Cu (II). After the reaction, the two peaks shifted to 943.98 eV and 935.57 eV, respectively. A new peak at 940.97 eV was observed after the reaction, suggesting the appearance of a new valence state which could be ascribed to Cu (I) [45]. The generation of Cu (I) illustrated that Cu (II) was reduced to a +1 valence state via the catalytic process, which was similar to PMS activation and could be explained by the coexisting oxidation and reduction processes (Cu^2+^-Cu^+^-Cu^2+^) [14]. Figure 4b shows the XPS spectrum of Fe 2p before and after the reaction. The binding energy at 718.97 eV and 725.75 eV can be assigned to Fe^2+^ and Fe^3+^, respectively [46]. Obviously, little to no redox reactions involving Fe^2+^/Fe^3+^ occurred on the CuFe_2_O_4_ surface, which was deduced because the Fe valence state hardly changed, as indicated by the Fe 2p_3/2_ and Fe 2p_1/2_ sites after the reaction. Similar results were also reported in previous studies [29,47]. Although PAA activation has been reported in homogeneous systems via the Fe^2+^/Fe^3+^ redox cycle, iron ions exhibited lower activity than copper ions. In heterogeneous systems, the contribution of the Fe^2+^/Fe^3+^ redox cycle was limited to the catalyst surface at neutral pH conditions [29]. The O 1s XPS spectrum shown in Figure 4c illustrates two major peaks at 530.38 eV and 531.54 eV before the reaction. These peaks were assigned to the lattice O and surface adsorbed oxygen (or surface hydroxyl species), respectively [48,49]. The proportions of lattice O and adsorbed oxygen (or surface hydroxyl species) before the reaction were 6.7% and 93.3%, respectively, while lattice O nearly disappeared after the reaction. It can be inferred that strong hydroxylation reactions happened on the catalyst surface during the oxidation process. The decrease of lattice oxygen was due to the reduction of Cu^2+^ to Cu^+^, and the increase of surface hydroxyl species could be attributed to the formation of Cu-OH complexes via Equations (13) and (14).

### 2.5. Identification of Reactive Species

**Figure 5:**
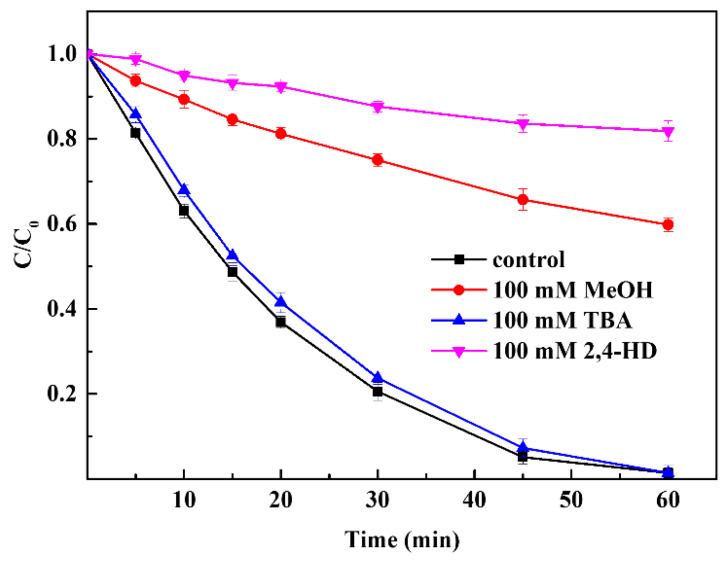
Effect of radical scavengers on Rhodamine B degradation (PAA = 80 mg/L, CuFe_2_O_4_ = 100 mg/L, pH = 7, C_0_ = 20 mg/L, room temperature for 1 h).

Several organic radicals (e.g., CH_3_COO˙, CH_3_COOO˙, CH_3_˙, and CH_3_CO_2_˙) and OH˙ have been reported in PAA activation AOP systems. To identify the free radicals that played a major role in the process, three kinds of radical scavengers (MeOH, PBA, and 2,4-hexadiene (2,4-HD)) were tested in the CuFe_2_O_4_/PAA processing system, as shown in Figure 5. TBA is a widely used OH˙ quencher with a second order rate constant of 3.8–7.6 × 10^8^ but zero-order with respect to acetyl(per)oxyl radicals [50]. When 100 mmol·L^−1^ TBA was added into the CuFe_2_O_4_/PAA system, the degradation of Rhodamine B was not impacted to a great extent. The final removal efficiency was 98.6% after 60 min of reaction, demonstrating a small difference from the condition with no TBA addition. This phenomenon suggested that OH˙ did not play an irreplaceable role in the degradation of Rhodamine B, which was consistent with the results of previous studies [51]. There were two C=C double bonds contained in 2,4-HD, which could be easily attacked by peroxyl and acylperoxyl radicals. Therefore, 2,4-HD was used as the quencher for acetyl(per)oxyl radicals [52]. When 100 mmol·L^−1^ 2,4-HD was added, the degradation of Rhodamine B after 60 min decreased from 98.6% to 18.2%. Obviously, acetyl(per)oxyl radicals played the dominant role in the CuFe_2_O_4_/PAA system for the degradation of Rhodamine B. The remaining oxidation capacity of 18.2% was attributed to the existence of OH˙. MeOH was a strong scavenger for both OH˙ and organic radicals [1]. When 100 mmol·L^−1^ MeOH was added into the CuFe_2_O_4_/PAA system, the degradation of Rhodamine B after 60 min decreased from 98.6% to 40.2%. According to the above conclusions, acetyl(per)oxyl radicals were the dominant radicals for degradation in the CuFe_2_O_4_/PAA system, whereas OH˙ played an ancillary role.

**Figure 6:**
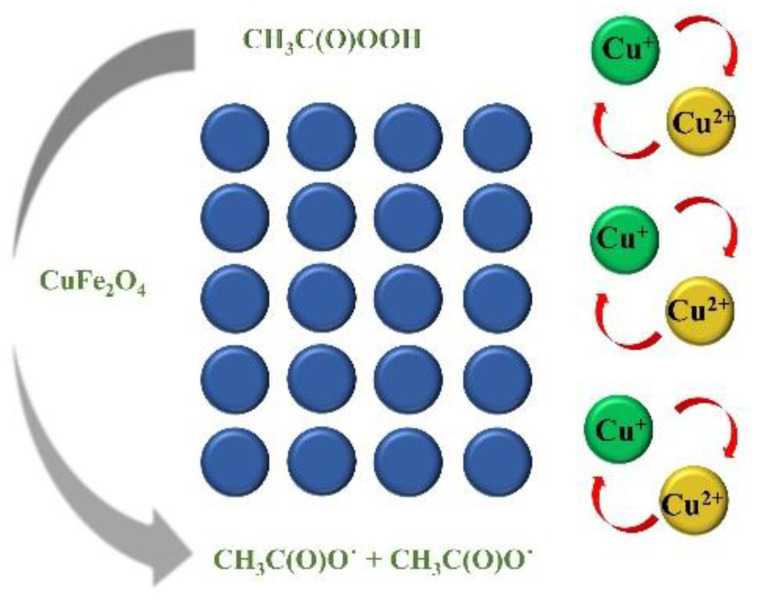
The proposed mechanism of the CuFe_2_O_4_/PAA system.

Based on the above discussion, a possible activation mechanism in CuFe_2_O_4_/PAA was proposed in Figure 6. The degradation of Rhodamine B was attributed to R-O˙ in the heterogeneous process. During the preliminary stage, ≡Cu^2+^ accepted an electron from PAA and was converted to ≡Cu^+^ which caused the subsequent generation of CH_3_C(O)OO˙. The generated ≡Cu^+^ donated an electron to PAA, resulting in the formation of CH_3_C(O)O˙ and the maintenance of the ≡Cu^2+^/ ≡Cu^+^ redox cycle on the surface of the catalyst.

≡Cu^2+^ + OH^−^ → ≡CuOH^+^ (13)

≡CuOH^+^ + OH^−^ → ≡Cu(OH)_2_ (14)

### 2.6. The Effect of Water Matrices on Contaminant Degradation

**Figure 7:**
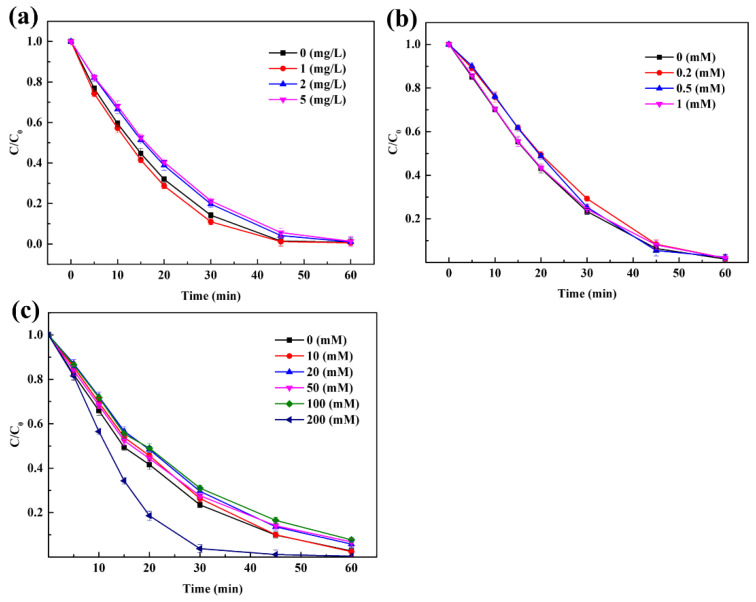
Effect of water matrices. (**a**) HA. (**b**) HCO_3_^−^. (**c**) Cl^−^ (PAA = 80 mg/L, CuFe_2_O_4_ = 100 mg/L, pH = 7, C_0_ = 20 mg/L, room temperature for 1 h).

Humic acid (HA), Cl^−^, and HCO_3_^−^ are pervasive substances in natural water which may suppress AOPs [53], so the effect of these naturally occurring anions on the degradation of Rhodamine B was further evaluated. HA could adsorb on the surface of the catalyst and block the catalyst active sites in heterogeneous systems to prevent contact between the oxidant and catalyst [53], resulting in the reduction of catalytic activation efficiency. In addition, R-O˙ could be partially scavenged by HA, which could also affect the pollutant degradation efficiency. However, the shielding effect was not obvious in the CuFe_2_O_4_/PAA system. The effect of HA on Rhodamine B degradation is shown in Figure 7a. Obviously, HA only had a slight impact on Rhodamine B removal in the CuFe_2_O_4_/PAA system. As the HA concentration increased from 0 mg/L to 5 mg/L, the removal efficiency after 60 min decreased from 99.2% to 98.7%. This may be because the added CuFe_2_O_4_ was in excess for PAA catalysis. In addition, HA may have pro-oxidant properties in AOPs [54]. Moreover, although the generated R-O˙ could be partially scavenged by HA, as demonstrated in previous studies [18], the remaining R-O˙ and other radicals such as OH˙ were abundant enough to lead to Rhodamine B removal.

The effect of HCO_3_^−^ on Rhodamine B degradation is shown in Figure 7b. HCO3^−^ had an ignorable impact on Rhodamine B removal in the CuFe_2_O_4_/PAA system. As the HCO_3_^−^ concentration increased from 0 mmol·L^−1^ to 1 mmol·L^−1^, the removal efficiency after 60 min decreased from 98.5% to 98.0%. It has been reported that HCO_3_^−^ is a scavenger of hydroxyl radicals and sulfate radicals but could not affect R-O˙ radicals [22]. Similar to the HA experiment, the disappearance of single free radicals did not affect the overall removal of pollutants. In addition, unreactive carbonate complexes [14,29] were not generated, suggesting the stability of the system. The effect of Cl^−^ on Rhodamine B removal is shown in Figure 7c. The degradation efficiency of Rhodamine B after 30 min was 76.5%, 72.4%, and 96.2% with 0-, 50-, and 200-mmol·L^−1^ Cl^−^ added, respectively. The final degradation efficiencies after 60 min were 97.3%, 93.2%, 99.6% with 0, 50, 200 mmol·L^−1^ Cl^−^ added, respectively. Apparently, the effect of Cl^−^ on the degradation of Rhodamine B was different under different Cl^−^ concentrations. A small dosage of Cl^−^ (10–100 mmol·L^−1^) suppressed the degradation of Rhodamine B while the addition of 200 mmol·L^−1^ Cl^−^ promoted the degradation of Rhodamine B. In addition, it was worth noting that the small dosage of Cl^−^ only slowed down the reaction speed while the final degradation was not seriously influenced. This was because Cl^−^ could react with R-O˙ and OH˙ to produce chlorine-containing species that had weaker oxidative capacities [55,56]. When 200 mmol·L^−1^ Cl^−^ was added, the generation of Cl, Cl_2_˙, and ClOH˙ compensated for the weakened oxidative capacity, and the radicals even synergized with the remaining R-O˙ and OH˙. In summary, water matrices had only a minimal negative effect on the application of the CuFe_2_O_4_/PAA system, which increased the possibility of the practical application of this type of AOP system.

### 2.7. Reusability and Stability of the Catalyst

**Figure 8:**
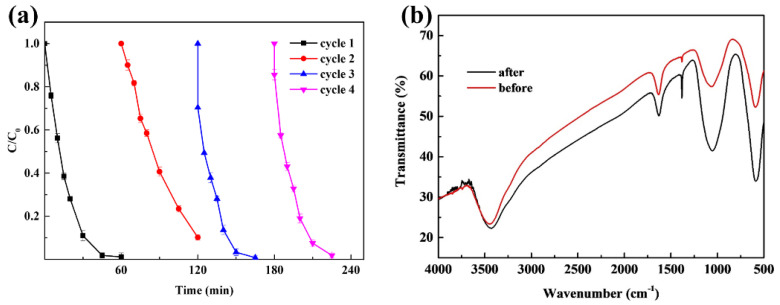
(**a**) Reusability of CuFe_2_O_4_ (PAA = 80 mg/L, CuFe_2_O_4_ = 100 mg/L, pH = 7, C_0_ = 20 mg/L, room temperature for 1 h). (**b**) FTIR spectra of CuFe_2_O_4_.

In terms of practical application, reusability and stability are very important characteristics of a catalyst. To evaluate the reusability of CuFe_2_O_4_, the removal of Rhodamine B by recycling the catalyst after the reaction concluded was conducted under the same experimental conditions (CuFe_2_O_4_ dosage of 100 mg/L, PAA dosage of 80 mg/L, initial pH of 7, and treatment time of 60 min) for four cycles, as shown in Figure 8a. The final removal efficiencies were 98.9%, 89.8%, 99.2%, and 98.2%, respectively, for the four iterative catalyst recycling experiments. It was obvious that CuFe_2_O_4_ demonstrated excellent catalytic performance, and the catalyst maintained a high performance for Rhodamine B decomposition after the fourth iteration of catalyst recycling. Only a 0.7% degradation efficiency decrease was observed, suggesting that CuFe_2_O_4_ possessed high reusability towards PAA activation. In addition, The FTIR spectra of the catalysts before and after the reaction are shown in Figure 8b. No additional functional group characteristic peaks were observed for the recycled CuFe_2_O_4_, illustrating that no passivation occurred on the CuFe_2_O_4_ surface compared with the fresh catalyst, and the catalyst exhibited good catalytic stability. These results suggested that the use of CuFe_2_O_4_ could be operated within a controllable cost window, owing to the high reusability and recyclability of the catalyst.

## 3. Materials and Methods

### 3.1. Chemicals

Commercial peracetic acid (~20% PAA, ~5% H_2_O_2_, and ~20% acetic acid; *w*/*w*) was obtained from Sigma-Aldrich (St. Louis, MO, USA). CuFe_2_O_4_ with a size of approximately 200 nm was supplied by Aladdin Co., Ltd. (Shanghai, China). Rhodamine B was supplied by Sigma-Aldrich (St. Louis, MO, USA). 2,4-hexadiene (2,4-HD),tert-butyl alcohol (TBA), methanol (MeOH), NaCl, Na_2_HCO_3_, Na_2_S_2_O_3_, and humic acid (HA) were purchased from Aladdin (Shanghai, China). All chemicals were of at least reagent grade and used without further purification. Nano-pure water (resistivity > 18 mΩ cm) was obtained from a Merck Milli-Q Reference system (Darmstadt, Germany).

### 3.2. Experimental Procedures

Batch degradation experiments were performed at room temperature (25 °C), and the solution was mixed by magnetic stirring in 250 mL amber glass bottles. After an appropriate amount of Rhodamine B solution (20 mg/L) was transferred into the reactor, PAA at designated concentrations (10–100 mg/L) was subsequently added. Then, the initial pH was adjusted by 0.2 mol·L^−1^ NaOH and 0.1 mol·L^−1^ H_2_SO_4_ solution. The experiments started as soon as the desired amount of CuFe_2_O_4_ was added. The samples were extracted at predetermined time intervals and immediately quenched with excess Na_2_S_2_O_3_ (10 mmol·L^−1^) for the detection of the Rhodamine B concentration. The quenched sample was filtered through a 0.22-μm PTFE membrane, and the filtered sample was subsequently stored at 4 °C before analysis within 24 h.

Cl^−^ (0–200 mmol·L^−1^), HCO_3_^−^ (0–1 mmol·L^−1^), and humic acid (HA, 0–5 mg/L) were added at the beginning of the reaction to evaluate the effect of water matrices on the degradation of Rhodamine B. The same reaction procedures were repeated with tertbutyl alcohol (TBA, 100 mmol·L^−1^), methanol (MeOH, 100 mmol·L^−1^), and 2,4-HD (100 mmol·L^−1^) added at the beginning of the reaction to assess the presence of radical species.

The used CuFe_2_O_4_ was collected via magnetic separation, washed with distilled water, and dried via a vacuum freeze dryer (FD-1A-50, Boyikang, Shanghai, China) to evaluate reusability. All experiments were conducted in duplicate.

### 3.3. Analytical Methods

N_2_ adsorption–desorption measurements were conducted using an ASAP 2020 volumetric adsorption analyzer (Micromeritics, Norcross, GA, USA). The specific surface areas were measured using the standard Brunauer-Emmett-Teller (BET) method. The surface morphology of the samples was characterized via a scanning electron microscope (SEM, ZEISS SUPRA 55, Jena, Germany). The composition and chemical oxidation state of the elements were determined by X-ray photoelectron spectroscopy (XPS, ESCALAB 250Xi, ThermoFisher Scientific, Waltham, MA, USA). Fourier-transform infrared spectroscopy (FTIR, VERTEX 70, Bruker, Karlsruhe, Germany) was used for the characterization of CuFe_2_O_4_ before and after the reaction. The pH values were measured via a pH meter (Starter-3100, Ohaus, Parsippany-Troy Hills, NJ, USA). The PAA concentration was measured according to the N, N-diethyl-p-phenylenediamine (DPD) colorimetric method [57]. The Rhodamine B concentration was measured via a UV-Vis spectrophotometer (Agilent, Santa Clara, CA, USA) at 553 nm.

## 4. Conclusions

PAA demonstrated promise as an efficiency disinfectant in AOPs. Spinel ferrite exhibited advantages in terms of structural stability and recovery, and Cu had a lower risk of toxicity and carcinogenicity. The current work demonstrated that the CuFe_2_O_4_/PAA system was a promising process for the degradation of organic pollutants. A pH range of six to eight was suitable for efficient degradation. As the loading of PAA or CuFe_2_O_4_ increased (10–100 mg/L for PAA and 0–100 mg/L for CuFe_2_O_4_), the oxidation of pollutants also increased. Taking into consideration cost control measures and treatment effects, an unlimited increase in dosage was undesirable and unrealistic. When the PAA dose was 80 mg/L and the CuFe_2_O_4_ dose was 100 mg/L, a Rhodamine B removal efficiency of 95.8% was observed, which was sufficient for sewage treatment. PAA activation was dominated by the ≡Cu^2+^/≡Cu^+^ redox cycle, and the pollutant was removed mainly by the oxidation of CH_3_C(O)O˙ and CH_3_C(O)OO˙. In terms of application in actual wastewater treatment scenarios, a small number of common water matrices (HA, HCO_3_^−^, and Cl^−^) had a small negative effect on the process, while 200 mg/L of Cl^−^ enhanced the oxidation process. In addition, the CuFe_2_O_4_/PAA system demonstrated satisfying reusability and stability after four recycling iterations. However, further studies are needed in terms of optimization and intensification before promotion and application can be achieved.

## Supplementary Materials

The following supporting information can be downloaded at: https://www.mdpi.com/article/10.3390/molecules27196385/s1, Figure S1: Rhodamine B degradation with and without PAA (PAA = 80 mg/L, pH = 7, C_0_ = 20 mg/L, room temperature for 1 h), Figure S2: The full scale XPS spectrum of CuFe_2_O_4_.

## Sample Availability

Samples of the Peracetic Acid and CuFe_2_O_4_ are available from the authors.
