# Supplementary material for: Activation of Peracetic Acid with CuFe2O4 for Rhodamine B Degradation: Activation by Cu and the Contribution of Acetylperoxyl Radicals
Source: Molecules. 2022 Sep 27;27(19):6385. doi: 10.3390/molecules27196385 (PMC9571141; doi:10.3390/molecules27196385)
Supplement: Supplementary file 1 [file molecules-27-06385-s001.zip › molecules-1899125-supplementary.pdf]

Supporting Information for

Activation of Peracetic Acid with $CuFe_2O_4$ for Rhodamine B Degradation: Activation by Cu and the Contribution of Acetylperoxyl Radicals

Chengzhi Yu [1], Libin Zheng [1], Yongyuan Hong [1], Jiabin Chen [1], Feng Gao [1,*], Yalei Zhang [1,2], Xuefei Zhou [1,2] and Libin Yang [1,*]

[1] State Key Laboratory of Pollution Control and Resource Reuse, College of Environmental Science and Engineering, Tongji University, Shanghai 200092, China

[2] Shanghai Institute of Pollution Control and Ecological Security, Tongji University, Shanghai 200092, China

* Correspondence: neuqyanglibin@126.com (L.Y.); gaofeng_1111@126.com (F.G.)

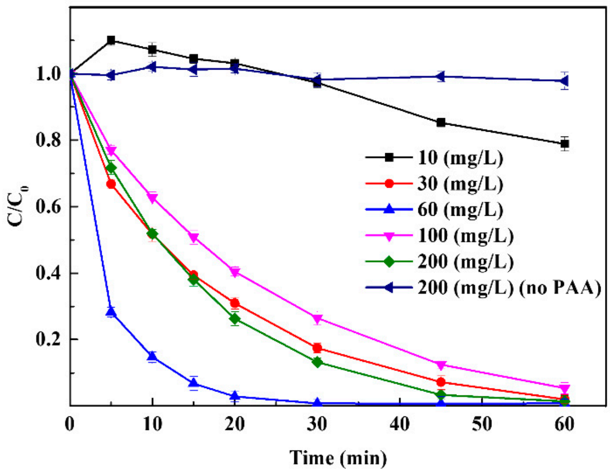

Figure S1. Rhodamine B degradation with and without PAA (PAA = 80 mg/L, pH = 7, C_0 = 20 mg/L, room temperature for 1 h).

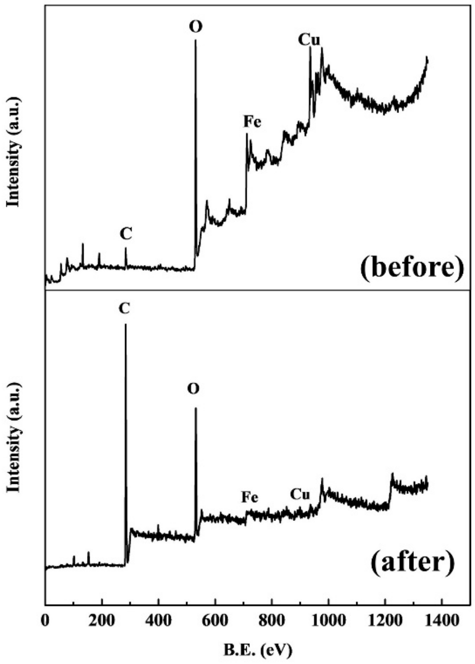

Figure S2. The full scale XPS spectrum of $CuFe_2O_4$.
